# A Clinical and Pathological Overview of Vulvar Condyloma Acuminatum, Intraepithelial Neoplasia, and Squamous Cell Carcinoma

**DOI:** 10.1155/2014/480573

**Published:** 2014-02-25

**Authors:** Boris Léonard, Frederic Kridelka, Katty Delbecque, Frederic Goffin, Stéphanie Demoulin, Jean Doyen, Philippe Delvenne

**Affiliations:** ^1^Department of Pathology, University Hospital of Liège, 4000 Liège, Belgium; ^2^Department of Gynecology, University Hospital of Liège, 4000 Liège, Belgium; ^3^Laboratory of Experimental Pathology, GIGA-Cancer, University of Liège, 4000 Liège, Belgium

## Abstract

Condyloma acuminatum, intraepithelial neoplasia, and squamous cell carcinoma are three relatively frequent vulvar lesions. Condyloma acuminatum is induced by low risk genotypes of human papillomavirus (HPV). Vulvar intraepithelial neoplasia (VIN) and squamous cell carcinoma have different etiopathogenic pathways and are related or not with high risk HPV types. The goal of this paper is to review the main pathological and clinical features of these lesions. A special attention has been paid also to epidemiological data, pathological classification, and clinical implications of these diseases.

## 1. Introduction

Vulvar human papillomavirus (HPV) infection is responsible for the development of benign tumors (condylomata acuminata), of one type of preneoplastic lesions, and of certain types of squamous cell carcinoma (SCC) [1]. Condylomata acuminate are vulvar exophytic benign tumors which result usually (90%) from HPV types 6 and 11 (several other HPV types can be involved) [2]. Up to 83% of women with external genital warts or a history of external genital warts have a concomitant cervical HPV infection [3].

Similarly to the cervix, most of the vulvar (pre)neoplastic lesions are induced by HPV infection (most commonly HPV 16), except for the “differentiated” (simplex) type of VIN.

This allows to distinguish two types of VIN: (1) the usual VIN (uVIN)/classic VIN (WHO terminology) which is related to HPV infection; (2) the differentiated/simplex type (dVIN), non-HPV-related, but associated with vulvar dermatoses, especially the lichen sclerosus [4, 5].

The distinction is also applicable for SCC with “HPV-related SCC,” associated with uVIN and “non-HPV-related vulvar SCC,” often associated with dVIN and lichen sclerosus [1, 4, 6].

The incidence of HPV-associated VIN, unlike that of vulvar carcinomas, has been increasing over the past 20 years, especially in women of reproductive age, with the highest frequency reported in women of 20–35 years old [7–9]. dVIN type and non-HPV-related vulvar SCC occur commonly in elderly women [9].

Approximately 95% of malignant tumors of the vulva are SCC. They represent 6.38% of all gynaecologic cancers in Belgium (Belgian Cancer Registry 2011). The American Cancer Society reports over 3,400 new cases of vulvar SCC in the USA annually [10].

The incidence of both types (HPV and non-HPV associated) of vulvar SCC increases with age [11, 12]. The reported incidence rates are 1 : 100,000 in younger women and 20 in 100,000 in the elderly [13]. The mean age at presentation is 60–74 years [7, 14]. Vulvar carcinoma may infrequently occur in younger women and adolescents [15, 16]. The peak incidence of vulvar cancer in Belgium (Belgian Cancer Registry 2011) is observed in people over the age of 85.

## 2. Condyloma Acuminatum (“Genital Warts”)

### 2.1. Clinical Features

Condyloma acuminatum (CA) or venereal/genital warts refer to benign proliferative epidermal or mucosal lesions attributed mostly to HPV type 6 or 11, but coinfections with high-risk HPV types are frequent. More than 100 types of HPV have been identified, of which 40 can infect the genital areas. HPV are highly specific viruses showing both species and regional specificity. They represent the most common sexually-transmitted disease (STD) and are highly contagious. The prevalence of CA peaks in the early sexually active years, with two-thirds of the respective sexual partners complaining of warts. The median time between infection and development of lesions is about 5-6 months among women. Up to 20% of people with genital warts will present other STDs.

The following risk factors have been described, including smoking, hormonal contraceptives, multiple sexual partners, and early coital age. Patients who develop CA complain of painless bumps and, less frequently, of pruritus, discharge, or bleeding. Lesions are commonly multiple (multicentric) and multifocal, also affecting the perianal, vaginal, and cervical regions, but oral and laryngeal regions may also be involved. Latent illness may become active, particularly with pregnancy and immunosuppression. Lesions may regress spontaneously, remain stable, or progress in size and/or number.

CA are soft, raised masses, with smooth, verrucous, or lobulated aspects that may appear as pearly, filiform, fungating, or plaque-like eruptions. The surface commonly shows finger-like projections, generally nonpigmented. They mainly occur in the moist areas of the labia minora and vaginal opening, but virtually, all genital regions may be affected (fourchette, labia minora/majora, pubis, clitoris, urethral meatus, perineum, perianal region, anal canal, introitus, vagina, and ectocervix). Therefore, minutious colposcopic examination, using acetic acid 2–5%, is of crucial importance to detect potentially multiple involved sites.

CA are perceived as disfiguring, they impact sexual lifestyle, causing anxiety, guilt, and loss of self-esteem and creating concerns about cancer risk.

The most common treatments are painful and nonspecific, addressing the clinically evident lesions rather than the viral cause. Various modalities include office-based treatment (cryotherapy, electrocautery, laser, and/or surgery) or home-based treatment (chemotoxic agents or immunomodulatory therapy). First episode patients should be STD screened. Management should include partner notification.

### 2.2. Etiopathogenesis

The initial site of infection is thought to be either basal cells of the immature squamous epithelium that HPV reaches presumably through defects in the epithelium. Once HPV enters the cells, two distinct biological sequences are possible. The first form is a “nonproductive or latent infection” in which HPV DNA persists in the basal cells without virus replication. Latent infections do not show morphologic alterations and can only be identified using molecular methods.

The second form of HPV infection is a “productive infection.” Viral DNA replication in the intermediate and superficial cell layers of the squamous epithelium occurs independently of host chromosomal DNA synthesis. This allows large amounts of intact virions to be formed, leading to typical morphological aspects such as “koilocytic changes.”

Molecular biologic methods have identified HPV-6 as the most common HPV type in CA. HPV-11 has been found in approximately one fourth of genital warts. These two HPV types are responsible for over 90% of CA [17, 18].

### 2.3. Pathologic Features

#### 2.3.1. Gross Findings

The lesions are typically exophytic and may range from discrete papillary excrescences to extensive and coalescent “cauliflower-like” masses [4].

#### 2.3.2. Microscopic Findings

**Figure 1:**
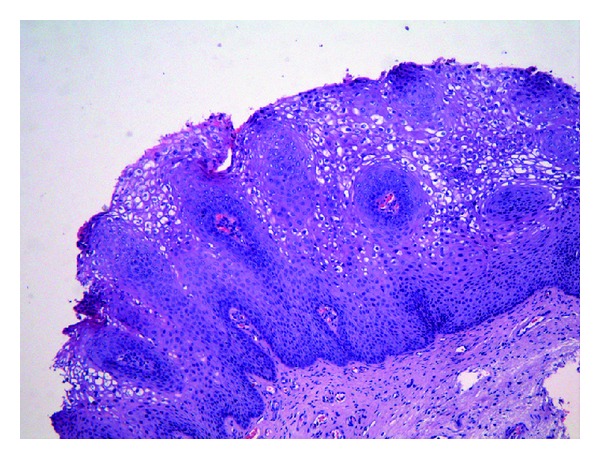
Vulvar condyloma acuminatum with acanthotic squamous epithelium and prominent koilocytic changes.

CA shows a striking papillary architecture. Papillae of different sizes and shapes are lined by acanthotic squamous epithelium and have a fibrovascular stroma, often containing scattered chronic inflammatory cells. Hyperkeratosis, parakeratosis, hypergranulosis, and basal cell hyperplasia are seen. Koilocytic changes (rigid perinuclear halos, binucleated nuclei, and slightly enlarged nuclei with irregular contours and coarse chromatin), sometimes focal, are present in the most superficial layers of the squamous epidermis. Mitotic figures are observed in the lower third of the epidermis (Figure 1) [1, 4].

#### 2.3.3. Ancillary Studies

The proliferation index by immunostaining (Ki67/MIB-1) in the upper third of the epithelium is considered as an adjunct test to confirm the diagnosis of CA, especially in lesions without evident koilocytic changes.

The presence of Ki67/MIB-1 immunostaining has been further correlated with the detection of HPV DNA by polymerase chain reaction (PCR).

In situ hybridization for HPV can also be performed on paraffin-fixed tissue sections to confirm the presence of HPV-DNA in CA. However, this test may lack sensitivity [4].

### 2.4. Treatment Options

#### 2.4.1. Home Therapy

- Podophyllotoxin (0.15% cream or 0.5% solution) is an antimitotic and cytotoxic molecule that results in necrosis of genital CA. Each course of podophyllotoxin treatment comprises self-application twice daily for 3 days, followed by four rest days. However, vulvar and anal warts are more feasibly and efficiently treated with clearance rates of 45–83% after use of 0.5% podophyllotoxin solution for 3–6 weeks. Transient and acceptable burning, tenderness, erythema, and/or erosions for a few days when the warts necrotize are described. Recurrence rates of 6–100% have been reported with podophyllotoxin between 8 and 21 weeks after clearance [19–21]. Podophyllotoxin is contraindicated during pregnancy, and women of childbearing age must use contraception.
- Imiquimod 5% topical cream (Aldara) induces interferon production and is a cell-mediated immune response modifier. It is applied to the warts three times a week at bedtime. Treatment continues until wart clearance, or for a maximum of 16 weeks. It presents minimal systemic absorption but causes erythema, irritation, ulceration, and pain at application site. In clinical studies, clearance has been reported in 35–68% of patients with treatment courses up to 16 weeks. Erythema is often seen as a side effect with imiquimod therapy and sometimes appears to precede clinical resolution. Occasionally, severe inflammation is seen necessitating discontinuation of therapy. Relatively low recurrence rates (6–26%) after successful clearance have been reported [13, 22, 23].
- 5-Fluorouracil: no longer recommended for routine use, it has antimetabolic and/or antineoplastic and immunostimulative activity.
- Sinecatechins ointment: Sinecatechins is available as a 10% ointment in Europe and a 15% ointment in the US. It consists in a preparation of green tea catechins (sinecatechins). Evidence suggests that the mechanism of action is made through antiproliferative mechanisms. The ointment is applied three times a day until complete clearance, or for up to 4 months. It cannot be used internally or during pregnancy. Clearance rates of 47–59% over 12–16 weeks were reported. Local side effects occur as frequently as with other topical therapies. They are generally graded as mild and typically include redness, burning, itching, and pain at the site of application. In those clearing, low recurrence rates of 7–11% were observed over 12 weeks follow-up [24–26].

#### 2.4.2. Office Therapy

- Trichloroacetic acid (TCA) solution (80–90%) is used directly to the wart surface, weekly. It rapidly penetrates and cauterizes skin, keratin, and other tissues. Although caustic, this treatment causes less local irritation and systemic toxicity and has low cost. Response rates of 56–81% have been reported, with recurrence rates of 36% [27]. TCA can be used safely during pregnancy.
- Cryotherapy may be performed weekly using an open spray or cotton-tipped applicator for 10–20 seconds and repeated as needed. A freeze-thaw-freeze technique is applied to each lesion at each session. Application techniques are difficult to standardize and there may be significant intraoperator differences. Cryotherapy presents the advantages of being simple, inexpensive, rarely causes scarring or depigmentation, and is safe during pregnancy. Clinical studies have reported clearance rates in the range of 44–75%, and recurrence rates of 21–42% one to three months after clearance [27–29].
- Surgical treatments have the highest primary clearance rates with initial cure rates up to 60–90%. They include electrosurgery, curettage, scissors excision, and laser therapy. Surgery may be used as primary therapy, and the majority of patients can be treated under local anaesthesia. When performed carefully, simple surgical approach leaves highly satisfactory cosmetic results. Clearance rates of 94–100% and 89–100% have been reported for electrosurgery and scissors excision, respectively, with recurrence rates of 19–29%. Formal surgery, under anaesthesia, is convenient for the removal of bulky warts, extensive warts, and anal/intra-anal warts.

Many patients either fail to respond to treatment or recur after adequate response resulting in patient dissatisfaction. Global recurrence rates exceed 50% after 1 year due to repeated infection from sexual contact, persistence of virus in surrounding skin, hair follicles, sites not adequately reached by the intervention used, or immunosuppression [27, 30–32].

### 2.5. Primary Prevention by Prophylactic Vaccine (Quadrivalent Gardasil)

A quadrivalent HPV vaccine is available for prevention of HPV-associated dysplasia and neoplasia including cervical cancer, precancerous genital lesions, and genital warts associated with HPV types 6, 11, 16, and 18. Vaccine efficacy is mediated by humoral immune responses following immunization. It is indicated for prevention of CA caused by HPV types 6 and 11 in boys, men, girls, and women aged 9–26 years. The very high efficacy of the quadrivalent HPV vaccine against HPV6/11 disease was reported in multiple randomised, controlled trials. There is now accumulating evidence that population-based quadrivalent HPV vaccination can result in dramatic declines in genital warts incidence and reduction in HPV6/11 burden [33–35].

## 3. Vulvar Intraepithelial Neoplasia

The term “vulvar intraepithelial neoplasia” (VIN) was endorsed by the International Society for the Study of Vulvar Disease (ISSVD) in 1986 to describe intraepithelial neoplastic proliferations of the vulvar epidermis [4]. Previously, other terms had been used to describe histologically similar lesions: “Bowen's disease,” “erythroplasia of Queyrat,” “bowenoid papulosis,” and “bowenoid dysplasia” [4]. In 2004, the terminology for squamous VIN was reviewed by the ISSVD that classified VIN in two groups: usual type (uVIN) and differentiated type (dVIN). uVIN type is predominant while dVIN accounts for a small proportion (<2–5%) of all VIN lesions [36, 37]. Both types of VIN have the potential to progress to vulvar cancer.

### 3.1. Clinical Features

Usual-type VIN (warty, basaloid, or mixed) occurs in young women. The incidence peaks at 45–49 years old, and has increased in recent years and has nearly doubled in the last decades, especially in young women. It is causally related to HPV infection. Other risk factors include smoking and immunosuppression. The lesions are frequently multifocal and have the potential to progress to invasive carcinoma.

Differentiated VIN affects older women, tends to be unifocal, and unicentric. It is not associated with HPV infection, but it is associated with vulvar dermatosis, mainly lichen sclerosus. It also has the potential to progress to invasive carcinoma. VIN is clinically important because the rate of progression to invasive SCC is reported to be as high as 80%, if left untreated [38].

The disease is asymptomatic in about 50% of cases. When symptomatic, the main complaints include itching, pruritus, pain, and dyspareunia. The appearance is variable from unique to multifocal lesions, flat, raised, or eroded, white, grey, red, or brown. After visual or colposcopic examination, such lesions should be biopsied for histological examination. A complete gynaecologic examination is of paramount importance to exclude any multicentric lesions that may affect the cervix, the vagina, and the anal region. The diagnosis of VIN can be subtle. To avoid delay, the physician must exercise a high degree of suspicion. Vulvar biopsy should be used liberally.

### 3.2. Etiopathogenesis

A part of vulvar carcinogenesis (for uVIN and HPV-related vulvar SCC) is superposable to cervical carcinogenesis. The viral oncoproteins E6 and E7 play a key role in the (pre)malignant transformation. After viral DNA integration in human host-cells, these viral oncoproteins E6 and E7 are overexpressed. Then, E6 degrades the tumor suppressor protein p53, therefore inducing the absence of cell cycle arrest [39]. Moreover, E7 induces an inactive retinoblastoma tumor suppressor gene product, resulting in hyperproliferation of host cells, with overexpression of the cell cycle-related marker p16 [39].

Based on their associations with cervical and anogenital cancers, a nonexhaustive number of anogenital HPVs (HPVs 16, 18, 31, 33, 35, 39, 45, 51, 52, 56, 58, 59, 66,…) have been classified by the International Agency for Research on Cancer (IARC) as oncogenic [40].

Among women infected with high-risk types of HPV, other factors such as smoking, immunosuppression, and long-term use of oral contraceptives can result in a doubling or tripling of risk for HSIL (“high grade squamous intraepithelial lesion”) and invasive cancer [41].

The HPV-independent pathway of vulvar SCC and dVIN is not well known. Genetic mutations in TP53 [42, 43] or PTEN [44] and epigenetic alterations such as hypermethylation of the MGMT, RASSF2A, or TSP1 gene promoters [45] have frequently been detected in dVIN and vulvar SCC, suggesting that the alteration of these genes contributes to vulvar carcinogenesis.

### 3.3. Pathologic Findings

#### 3.3.1. Gross Findings

Typical low-grade VIN appears as pale areas, whereas high-grade VIN lesions appear as white or erythematous papules or macules that frequently coalesce or show a verrucous growth. Approximately 10–15% of the lesions are hyperpigmented.

**Figure 2:**
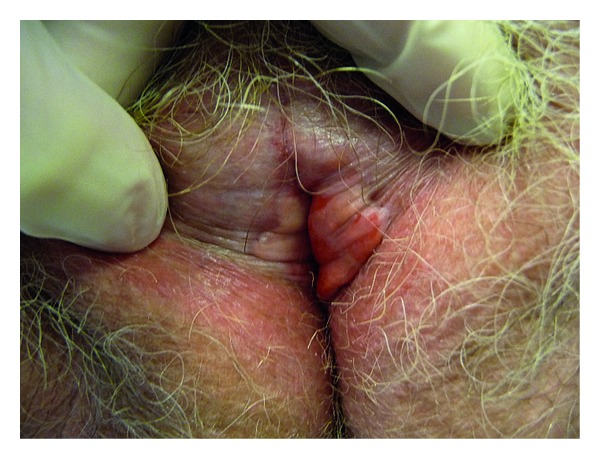
Differentiated VIN developed on sclerous lichen: white and surelevated nodules.

Differentiated/simplex VIN can be seen as focal discoloration, ill-defined white plaques, or discrete elevated nodules but they are typically less bulky than uVIN lesions [4]. Approximately two thirds of VIN lesions are multifocal (Figure 2) [1].

#### 3.3.2. Microscopic Findings

*(1) Usual/Classic VIN (uVIN). *uVIN shows morphological characteristics similar to all HPV-associated intraepithelial lesions such as cervical intraepithelial neoplasia (CIN), anal intraepithelial neoplasia (AIN), vaginal intraepithelial neoplasia (VaIN), and penile intraepithelial neoplasia [46].

These preneoplastic lesions are characterized by epithelial thickening and surface hyperkeratosis and/or parakeratosis. Dysplastic squamous cells with scant cytoplasm and hyperchromatic nuclei are accompanied by dyskeratotic cells with eosinophilic cytoplasm. Nuclear pleomorphism and hyperchromasia are present. However, nucleoli are uncommon. Loss of cell maturation and increased mitotic activity, including abnormal mitotic figures, are also seen [1, 4, 5].

uVIN involves the skin appendages in more than 50% of the cases studied [1]. uVIN is divided into warty (“condylomatous”) and basaloid types, essentially based on the architecture and appearance of the intraepithelial lesions.

The warty type shows a striking papillary pattern, acanthosis, with cytological signs of viral infection (koilocytic changes, multinucleation, and coarse granules) [1, 4].

The basaloid type presents a flat surface and is composed of a homogeneous population of small atypical parabasal type cells on nearly whole thickness of the epidermis. The epithelium lacks cellular maturation and koilocytic changes which are rarely seen [1, 4]. Sometimes, these two types are present within the same lesion [1, 4].

A rare variant is “pagetoid VIN” where atypical squamous cells present a pale cytoplasm and are isolated or grouped in small clusters [47, 48].

Based on the level of involvement of the thickness of the epithelium by the dysplastic cells, uVIN were graded in 3 grades (WHO terminology [5]):

- low-grade (VIN 1) if the dysplastic cells involve the lower third of the epithelium;
- moderate grade (VIN 2) when the dysplastic cells are present in the lower two-thirds of the epithelium;
- high-grade (VIN 3) if there is full-thickness involvement of the epithelium by the dysplastic cells. VIN 3 is synonymous with carcinoma in situ.

**Figure 3:**
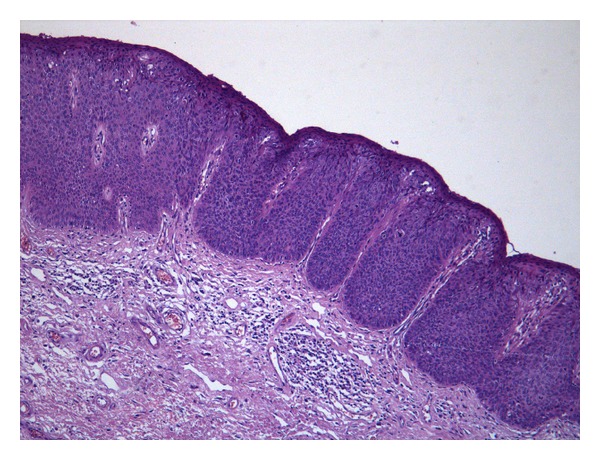
uVIN 3, basaloid type composed of a homogeneous population of dysplastic parabasal type cells on nearly whole thickness of the epidermis.

It is interesting to note that VIN 2 and VIN 3 confer the same risk and rate of progression to invasive carcinoma if untreated (Figure 3) [4].

The International Society for the Study of Vulvovaginal disease (ISSVD) has proposed that VIN should not be graded but described as high-grade VIN lesions only (VIN 2 or VIN 3). The ISSVD has also recommended that the term low-grade VIN (VIN 1, or mild dysplasia) should not be used anymore and that such lesions should be classified as flat condyloma acuminatum, or given an appropriate descriptive term [49].

*(2) Differentiated VIN (dVIN).* Differentiated (simplex) VIN is classified as high-grade VIN (thus VIN 3) due to its associated high risk to progress into invasive SCC [1, 4].

It shows epidermal hyperplasia with associated parakeratosis, with elongated and branched rete ridges. An important feature is the finding of squamous cells with abundant bright eosinophilic cytoplasm and typically prominent intercellular bridges. These keratinocytes, present in the basal as well as mid-layers of the epithelium, also show marked cytologic abnormalities such as large vesicular nuclei with macronucleoli.

**Figure 4:**
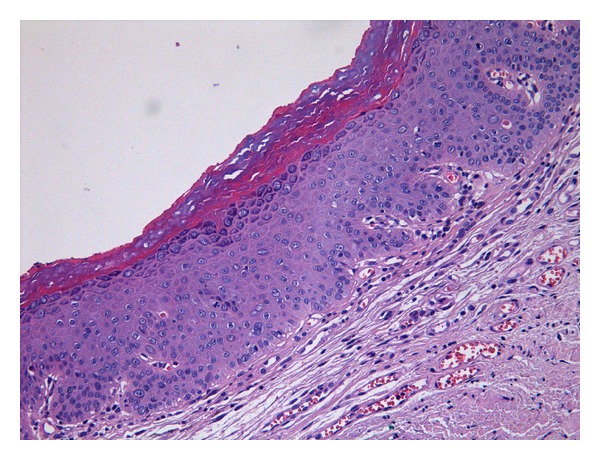
Differentiated VIN: atypical keratinocytes (with large vesicular nuclei with macronucleoli), present in the basal as well as mid layers of the epithelium. No koilocytic changes are identified.

Mitotic activity is more frequent at the base of the epidermis. No koilocytic changes are identified (Figure 4) [1, 4].

This lesion is frequently associated with lichen sclerosus and other cutaneous inflammation such as lichen simplex chronicus [42, 47].

#### 3.3.3. Ancillary Studies

p16, a surrogate marker of HPV, can be used to detect HPV infection. Diffuse and intense nuclear and cytoplasmic staining typically correlates with high-risk HPV infection. Focal and weak positivity is nonspecific. p16 immunostaining is characteristically negative in the epidermis of dVIN [50, 51].

Detection of increased proliferative activity in the upper layers of the epithelium using Ki67/MIB-1 staining has shown to be well correlated with the presence of HPV DNA by molecular analysis. Ki67/MIB-1 can help for distinction between dVIN and normal vulvar epidermis [52]. HPV in situ hybridization can also be used and is more specific than MIB-1 staining. However, this test suffers from low sensitivity [4].

PCR analysis of VIN 1 lesions has shown a mixture of low- and high-risk HPV (geno)types, whereas VIN 2 and VIN 3 are generally associated with high-risk HPV, most commonly HPV 16 and 18. Molecular studies have failed to demonstrate HPV DNA in dVIN. To identify dVIN, p53 staining can be used. 90% of dVIN show a high p53 positivity in the basal layer with suprabasal extension [42, 47].

### 3.4. Treatment Options

Due to the natural history of VIN, all VIN lesions should receive treatment. There is little consensus regarding the optimal method of management.

- The mainstay of management for VIN has been the surgical excision. One important advantage of surgical excision is that a complete histologic assessment is performed to exclude or define the presence of occult invasive carcinoma. The goal of the surgery is to obtain a 5-mm disease-free margin to control symptoms and to avoid malignant transformation. Results are initially good, but the recurrence rate is in between 30% to 50% [53]. Large and/or iterative ablations can lead to severe anatomical and functional sequellae, which is particularly distressing in young women. Because of this, and particularly because of the increase in younger women affected, great interest has been paid to nonsurgical treatment of VIN.
- Laser ablative therapy is an alternative to excision. The disadvantage of ablative therapy is that a necrotic ulcer on the vulva may result and wound healing may be slow. Complete healing may take several weeks. Pain, which is severe in some patients, is the main complication with laser therapy. Bleeding and infection have also been reported. The cosmetic results appear to be excellent. Laser therapy is an acceptable treatment modality, if invasive carcinoma has been ruled out. Many consider laser therapy the treatment of choice in the management of VIN, particularly for those who have multifocal disease. In the review article of the 253 patients treated with laser, 23% recurred [53].
- Since most uVIN lesions are associated with high risk-HPV types 16–18 infection, it has been hypothesized that the high recurrence rate following excision or ablative therapies is due to failure to remove the reservoir of HPV present in adjacent vulvar skin. Imiquimod is a low molecular weight imidazoquinoline acting as an immune response modifier, which could have the ability to generate HPV-specific cell-mediated immunity and potentially induce a regression of VIN lesions. Several studies have demonstrated that imiquimod is effective and safe for the treatment of VIN. Imiquimod 5% cream has been approved for the treatment of external anogenital warts and has shown safety and efficacy for different dermatological conditions such as external anogenital warts, superficial basal cell carcinoma, actinic keratosis, and extra genital Bowen's disease. In recent years, randomized control trials have shown that the application of 5% imiquimod is effective in the treatment of high-grade VIN [54]. The first studies had a short term follow-up, so presenting important bias because of the high risk of recurrence even after several years from the primary treatment. Recently, Terlou et al. published a report with seven-year median follow-up showing that in case of complete response, imiquimod is effective in the long-term [55]. However, all the investigations compared the patients treated with imiquimod to a control group treated with placebo, and only few authors analyzed data about the main outcomes in women treated with imiquimod and in women treated with different modalities. Imiquimod seems therefore to offer two important benefits: the avoidance of surgery and a lower recurrence rate for complete responders.

The risk factors for VIN recurrence after treatment are smoking, large lesions sizes, surgical specimen with positive margins. Because of the high risk of recurrence and risk of progression to invasive carcinoma, long-term follow-up is mandatory. The ACOG recommends an after therapy visit at 6 and 12 months, and then annually.

### 3.5. Primary Prevention

Recent randomized controlled trials have demonstrated that sustained protection from VIN can be offered with a prophylactic HPV vaccine. Immunization with HPV vaccination (bi or quadrivalent vaccine) has the potential to prevent about 70% of the VIN.

For example, the quadrivalent vaccine against HPV 6, 11, 16, and 18 was shown to be 97% effective in preventing VIN 2-3 in a population that was naive to these viruses at the time of first vaccination and 100% effective in those who remained unexposed through the completion of the vaccine regimen [56].

It seems that vaccinating HPV-naive women is efficacious, and it would be preferable to vaccinate women before they become sexually active.

## 4. Invasive Squamous Cell Carcinoma

### 4.1. Pathologic Features

#### 4.1.1. Gross Findings

**Figure 5:**
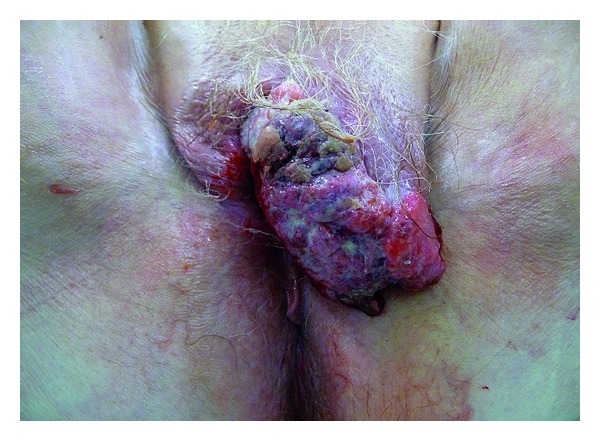
Exophytic and ulcerated squamous cell carcinoma.

SCC may appear as an exophytic or an endophytic ulcerated lesion. The labia majora and minora are preferentially involved. The majority of vulvar SCC are solitary. However, multifocal tumors are seen in 10% of cases (Figure 5) [4]. Clinical symptoms are usually related to the ulceration of the lesion. There is in average 12 to 18 months delay between initial symptoms and definitive histological diagnosis due to prescription of corticoid or antifungal topical therapy without detailed examination of the genitalia.

#### 4.1.2. Microscopic Findings

**Figure 6:**
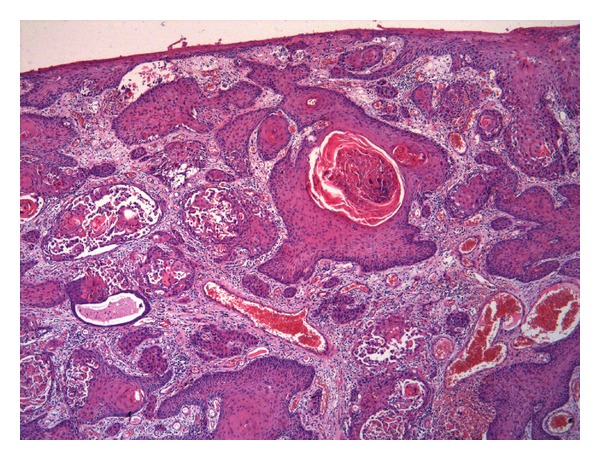
Keratinizing squamous cell carcinoma: infiltrative neoplastic cells are mature with abundant eosinophilic cytoplasm and show keratin pearls.

The current World Health Organization (WHO 2003) classification of vulvar tumors describes several variants of invasive squamous carcinoma [5]:

*(a) Keratinizing Squamous Cell Carcinoma NOS (Not Otherwise Specified).* This is the most common histologic subtype of SCC. Neoplastic cells are mature with abundant eosinophilic cytoplasm and show keratin pearl. The nuclei are enlarged with prominent nucleoli and features readily identified in most cases include considerable nuclear atypia and mitotic activity (Figure 6) [1, 4, 5]. Previously, keratinizing neoplasms are considered as non-HPV-associated tumors. The presence of dVIN and/or lichen sclerosus in adjacent skin was an evidence of an HPV-independent implication [50].

Recently, typing by “polymerase chain reaction” showed a significant number of discrepancies: prevalence of HPV in keratinizing SCC is observed up to 49.1% [57].

*(b) Nonkeratinizing Squamous Cell Carcinoma. *The cells in this subtype of invasive SCC show minimal evidence of keratinization with scattered keratinized cells but lacks keratin pearl. Keratin pearl formation is not observed [1, 4, 5]. Prevalence of HPV in nonkeratinizing SCC is 85.7% [57].

*(c) Basaloid Carcinoma.* This tumor subtype arises in association with high-grade uVIN and comprises irregular nests and cords of “basaloid” cells with scant cytoplasm. The cells are ovoid, relatively uniform in size, and the nuclei show evenly distributed granular chromatin with no evident nucleoli. No keratin pearls are observed [1, 4, 5]. Prevalence of HPV in basaloid SCC is 92.3% [57]. HPV-16 can be detected in approximately 70% of cases [1, 58]. 

*(d) Warty (Condylomatous) Carcinoma. *This histologic subtype is architecturally characterized by the presence of multiple papillary projections with fibrovascular cores. The papillae are lined by keratinized squamous epithelium showing koilocytic changes (the most characteristic features). Keratin pearl formation in the invasive nests is often seen [1, 4, 5, 59, 60]. Prevalence of HPV in warty carcinoma is 78.2% [57]. HPV 16 is frequently observed [59, 60].

*(e) Verrucous Carcinoma.* This highly differentiated variant of SCC is characterized by bulbous pegs of neoplastic cells that appear to push into the underlying stroma. The neoplastic squamous epithelium is hyperplastic and associated with prominent hyper- and parakeratosis. The tumor cells have abundant cytoplasm. Nuclear atypia is minimal. Mitotic figures are rare and koilocytosis is usually absent. Verrucous carcinoma presents no or very little metastatic potential. HPV type 6 has been identified in a number of verrucous carcinoma [4, 5, 61–64], but it is controversial in the literature; a recent retrospective study does not support a causal role of HPV in the development of verrucous carcinoma [65]. 

*(f) Squamous Carcinoma with Tumor Giant Cells.* This is a rare and aggressive variant of invasive SCC characterized by the presence of numerous multinucleated tumor giant cells. Large atypical nuclei with prominent nucleoli and brisk mitotic activity are frequent [4, 5].

*(g) Keratoacanthoma-Like Carcinoma.* This variant has been included in the last WHO classification (2003) of vulvar SCC. It presents an appearance of keratoacanthoma and occurs on hair-bearing skin. Histologically, it is characterized by the presence of a central crater filled with proliferating squamous epithelium and anucleated masses of keratin. Invading nests and cords of squamous epithelium are observed in the dermis. These tumors may regress spontaneously by a poorly understood immune mechanism [4, 5].

There is no grading system unanimously accepted for vulvar SCC. The American Joint Committee on Cancer (AJCC 2010) recommends a four-grade system: well-differentiated (G1), moderately differentiated (G2), poorly differentiated (G3), and undifferentiated (G4) (GX, grade cannot be assessed) [66].

The grading system recommended by the Gynecologic Oncology Group (GOG) is based on the percentage of undifferentiated cells (small cells with scant cytoplasm infiltrating the stroma).

Grade 1 tumors have no undifferentiated cells, Grade 2 tumors contain less than 50% undifferentiated cells, Grade 3 tumors have greater than 50% but less than 100%, and grade 4 is essentially entirely composed of undifferentiated cells. The risk of recurrence has been reported to be higher with increasing grade [67–69].

#### 4.1.3. Ancillary Studies

Immunohistochemical expression of p16 is commonly expressed in vulvar squamous tumors and VIN associated with oncogenic HPV [70, 71]. Indeed, the sensitivity and specificity of p16 immunostaining are close to 100% for detecting HPV-related carcinomas [50, 72]. Therefore, the anti-p16 antibody may be used as a good alternative to PCR [50, 73]. p53 staining is positive in 50–70% of HPV-unrelated vulvar SCC, which contrasts with a negativity of p53 staining in almost HPV-related vulvar SCC [50].

### 4.2. Treatment Options

Vulvar carcinoma benefits from a surgical staging system based on criteria established by the International Federation of Gynecology and Obstetrics (FIGO) [74]. The latter includes variables related to the primary disease (early or locally advanced stage) and the nodal status (negative versus ipsilateral or bilateral positivity).

Pretreatment assessment includes confirmation biopsy and careful perineal, vulvar, and vaginal examination. MRI and PET CT are not part of a routine work up and must be prescribed on individual basis.

Early stage carcinoma is best treated by a radical local excision with macroscopic tumor-free margins of 1 cm [75]. The “traditional” radical vulvectomy is no longer systematically applied due to its major deleterious impact on vaginal function. In the same operating time, nodal staging is justified when tumor depth exceeds 1 mm. Bilateral radical inguinofemoral (IF) nodal dissection carries a heavy potential morbidity (lymphocele, lymphoedema) and must therefore be individualized [76]. The sentinel node approach is nowadays considered validated for early stage neoplasms with a greater diameter <2 cm [77]. For early stage disease >2 cm, IF nodal staging must include superficial inguinal and deep femoral node dissection [78]. The procedure may be carried out ipsilaterally in case of labia major lateralised disease. The dissection must be bilateral in case of midline disease (minor labia, periclitoral, periurethral, or perianal) [79].

Locally advanced vulvar carcinoma based on vaginal, urethral, or anal involvement is treated by concomitant chemoradiation associating external beam, brachytherapy implant, and radiosensitizing platinum chemotherapy. In this context, nodal staging may precede the initiation of the radiotherapy [80]. External beam target volume may then be tailored on the basis of the patient's nodal status and limit the radiation induced morbidity in the absence of nodal metastases.

Recommended follow-up includes clinical vulvar, vaginal, and nodal examination on a three to six monthly basis. Indication of routine morphological or metabolic imaging exams must be individualized.

## 5. Conclusion

The understanding of all the aspects of these three relatively frequent diseases that are “vulvar condyloma acuminatum, vulvar intraepithelial neoplasia, and vulvar SCC” guarantees an optimal care of the patients. A close cooperation between clinicians and pathologists is also essential concerning their accurate diagnosis.
